# Spermidine Synthase (SPDS) Undergoes Concerted Structural Rearrangements Upon Ligand Binding – A Case Study of the Two SPDS Isoforms From *Arabidopsis thaliana*

**DOI:** 10.3389/fpls.2019.00555

**Published:** 2019-05-07

**Authors:** Bartosz Sekula, Zbigniew Dauter

**Affiliations:** Synchrotron Radiation Research Section, Macromolecular Crystallography Laboratory, National Cancer Institute, Argonne, IL, United States

**Keywords:** polyamine biosynthesis, triamine, spermine, spermidine, putrescine, decarboxylated S-adenosylmethionine, cyclohexylamine, aminopropyltransferase

## Abstract

Spermidine synthases (SPDSs) catalyze the production of the linear triamine, spermidine, from putrescine. They utilize decarboxylated S-adenosylmethionine (dc-SAM), a universal cofactor of aminopropyltransferases, as a donor of the aminopropyl moiety. In this work, we describe crystal structures of two SPDS isoforms from *Arabidopsis thaliana* (*At*SPDS1 and *At*SPDS2). *At*SPDS1 and *At*SPDS2 are dimeric enzymes that share the fold of the polyamine biosynthesis proteins. Subunits of both isoforms present the characteristic two-domain structure. Smaller, N-terminal domain is built of the two β-sheets, while the C-terminal domain has a Rossmann fold-like topology. The catalytic cleft composed of two main compartments, the dc-SAM binding site and the polyamine groove, is created independently in each *At*SPDS subunits at the domain interface. We also provide the structural details about the dc-SAM binding mode and the inhibition of SPDS by a potent competitive inhibitor, cyclohexylamine (CHA). CHA occupies the polyamine binding site of *At*SPDS where it is bound at the bottom of the active site with the amine group placed analogously to the substrate. The crystallographic snapshots show in detail the structural rearrangements of *At*SPDS1 and *At*SPDS2 that are required to stabilize ligands within the active site. The concerted movements are observed in both compartments of the catalytic cleft, where three major parts significantly change their conformation. These are (i) the neighborhood of the glycine-rich region where aminopropyl moiety of dc-SAM is bound, (ii) the very flexible gate region with helix η6, which interacts with both, the adenine moiety of dc-SAM and the bound polyamine or inhibitor, and (iii) the N-terminal β-hairpin, that limits the putrescine binding grove at the bottom of the catalytic site.

## Introduction

Putrescine (PUT), a linear diamine, is produced by plants from agmatine or ornithine. Agmatine route, which originated in plants by the horizontal gene transfer from the cyanobacterial ancestor of the chloroplast (Michael, 2017), involves three enzymes: arginine decarboxylase (ADC), agmatine iminohydrolase (AIH), and N-carbamoylputrescine amidohydrolase (CPA). The first enzyme of the pathway, pyridoxal 5′-phosphate-dependent ADC, converts arginine to agmatine. Later, agmatine is hydrolyzed to N-carbamoylputrescine by AIH, a dimeric enzyme with a 5-bladed propeller fold (Sekula and Dauter, 2019). The last step of the pathway that provides PUT involves the action of an octameric CPA with a characteristic subunit arrangement that resembles an incomplete helix (Sekula et al., 2016). The alternative route for PUT biosynthesis, ornithine pathway, is carried out by ornithine decarboxylase (ODC). However, this enzyme is not common for all plant species and some plants lack ODC relying only on agmatine biotransformation (Hanfrey et al., 2001).

Longer polyamines are produced by aminopropyltransferases (APTs), enzymes that catalyze the transfer of an aminopropyl group from decarboxylated S-adenosylmethionine (dc-SAM), a universal cofactor of APTs, to a polyamine substrate. In plant organisms, different polyamines are produced by distinct specialized APTs (Shao et al., 2012). Thus, spermidine synthase (SPDS) utilizes PUT as the substrate for spermidine (SPD) biosynthesis. Spermine and thermospermine are produced from SPD by spermine synthase (SPMS) and thermospermine synthase (TSPS), respectively. Although both APTs use the same substrate they present significant differences that predestine them to produce distinct products (Sekula and Dauter, 2018). From the group of plant APTs, only the structures of TSPS from *Medicago truncatula* (*Mt*TSPS, PDB ID 6bq2) (Sekula and Dauter, 2018), and SPDS1 from *Arabidopsis thaliana* (*At*SPDS1, PDB ID 1xj5, Center for Eukaryotic Structural Genomics) are known, however there is no published structural characterization of *At*SPDS1.

Polyamines are abundant cationic compounds that play a critical role in the growth and development of plants (Tiburcio et al., 2014). Increased stress tolerance of plants is one of the most important roles assigned to polyamines (Bouchereau et al., 1999). They take part in the regulation of stress signaling pathways (Kasukabe et al., 2004), scavenging of the reactive oxygen species (Radhakrishnan and Lee, 2013; Kamiab et al., 2014; Mostofa et al., 2014) and stabilization of the photosynthetic apparatus (Hu et al., 2014). Levels of the polyamines undergo massive changes in plants upon the biotic stress (Jiménez-Bremont et al., 2014) and their exogenous application may increase the resistance to pathogen infection (Gonzalez et al., 2011). Moreover, polyamines modulate the rate of membrane transport (Pottosin and Shabala, 2014; Pottosin et al., 2014), as well as they interact with nucleic acids and proteins, thus they take part in the regulation of the transcription and translation (Gill and Tuteja, 2010; Igarashi and Kashiwagi, 2010; Tiburcio et al., 2014). Dysfunctions of the polyamine biosynthesis pathway cause growth retardation, sterility, and other pathologies (Hanzawa et al., 2000). Polyamines are used to create conjugates with hydroxycinnamic acids to form hydroxycinnamic acid amides, essential compounds for certain developmental processes (Tiburcio et al., 2014) and precursors of defensive compounds (Burhenne et al., 2003). SPD plays an essential role in the hypusination process. It acts as a donor of the aminobutyl moiety for the posttranslational modification of lysine residue of the translation factor eIF5A. eIF5A participates in translation elongation and in plants is important for the control of flowering time, the aerial and root architecture, root hair growth, and adaptation for challenging growth conditions (Belda-Palazón et al., 2016).

Spermidine synthases most likely originated from the common ancestor before the separation of prokaryotes and eukaryotes. Then, independently in plants, fungi, and animals, they probably duplicated and evolved to acquire SPMS activity (Minguet et al., 2008). Another enzyme that probably originated from SPDS is PUT *N-*methyltransferase (Hashimoto et al., 1998a). Interestingly, the dc-SAM binding motifs of plant SPDSs are more homologous to this enzyme than to mammalian or bacterial SPDSs (Hashimoto et al., 1998b). On the other hand, TSPS originated in plants from the horizontal gene transfer, similarly to the proteins from the agmatine route. Therefore, it is not surprising that TSPSs show clear divergence from other APTs of the flowering plants (Sekula and Dauter, 2018), while SPDS and SPMS are more similar. *A. thaliana*, similarly to many other dicots, has two gene paralogs encoding SPDS1 and SPDS2 whose distribution differs across tissues, stage of development and environment (Alcázar et al., 2010). SPDS1, SPDS2, and SPMS in *A. thaliana* present dual subcellular localization and are localized in the nucleus and cytosol (Belda-Palazon et al., 2012). They can interact with each other to form heteromultimers (Panicot et al., 2002), however, these are found only in the nucleus. SPDSs can be potently inhibited by CHA and its cyclic or aromatic derivatives (Shirahata et al., 1991). The application of CHA leads to accumulation of the free polyamines and may stimulate radicle emergence and the miotic index (Gallardo et al., 1994).

In this work, we present a structural comparison of the two SPDS isoforms from *A. thaliana* (*At*SPDS1 and *At*SPDS2). We also discuss the binding mode of dc-SAM, the inhibition of *At*SPDS by CHA and the structural rearrangements of *At*SPDS upon the ligand binding.

## Materials and Methods

### Cloning, Overexpression, and Purification of *At*SPDS1 and *At*SPDS2

Complementary DNA (cDNA) of *A. thaliana* was obtained according to the protocol described earlier (Sekula et al., 2018). The cDNA was used as a template for a polymerase chain reaction in order to isolate *At*SPDS1 and *At*SPDS2 open reading frames (ORF), which are annotated in the GenBank as AJ251296.1 and AJ251297.1, respectively. Two sets of forward primers were designed in a way to clone complete ORFs and the truncated ORFs. Complete ORF of *At*SPDS1 was isolated with the following primers: TACTTCCAATCCAATGCCATGGACGCTAAAGAAACCTCT GCCA (forward) and TTATCCACTTCCAATGTTATCAATTGG CTTTTGACTCAATGACCTTCTT (reverse). In case of *At*SPDS2 these were: TACTTCCAATCCAATGCCATGTCTTCA ACACAAGAAGCGTCTGTTA (forward) and TTATCCA CTTCCAATGTTACTAGTTGGCTTTCGAATCAATCACCTTC (reverse). The second variant of *At*SPDS1 was isolated with the use of the same reverse primer and a different forward primer (TACTTCCAATCCAATGCCAAAAAGGAACCTGCTTGTTTC TCCACTG) which allowed to clone the gene starting from codon 34. Forward primer for the isolation of the second *At*SPDS2 variant starting from the codon 39 was as follows: TACTTCCAATCCAATGCCAAGGAGCCTTCTTGTATGTCCT CTATTATT. The amplification products were incorporated into a pMCSG68 vector (Midwest Center for Structural Genomics) according to the ligase-independent cloning protocol (Kim et al., 2011). Then, BL21 Gold *Escherichia coli* competent cells (Agilent Technologies) were transformed with the vectors which carried *At*SPDS1 and *At*SPDS2 genes and the truncated constructs. The proteins were overexpressed with N-terminal His_6_-tag followed by the tobacco etch virus (TEV) protease cleavage site and Ser-Asn-Ala linker, which is not cleaved from the expressed proteins. The cells were cultured at 37°C in LB medium with ampicillin at 150 μg/ml concentration until OD_600_ reached value 0.9 and then the culture was cooled to 10°C for 1 h. The culture was induced with 0.5 mM of isopropyl-β-D-thiogalactopyranoside, and the overexpression was carried out at 18°C for the next 16 h. Afterward, the cells were cooled to 4°C and were pelleted by centrifugation at 3,500*g* for 20 min. The supernatant was discarded and 35 ml of the binding buffer [50 mM HEPES pH 7.4; 500 mM NaCl; 20 mM imidazole; 1 mM tris(2-carboxyethyl)phosphine, TCEP] was added to the cell pellets in order to resuspend them before freezing at -80°C. The cells were then thawed and subjected to sonication in an ice/water bath. The total time of sonication was 4 min and it consisted of 4-s sonication bursts with the intervals of 26 s. Then, after centrifugation at 25,000*g* for 30 min at 4°C, the supernatant was separated from the cell debris by decantation and applied on the column packed with 5 ml of HisTrap HP resin (GE Healthcare) which was connected to Vac-Man (Promega). The resin with captured proteins was washed five times with 40 ml of the binding buffer. *At*SPDS1 and *At*SPDS2, still carrying the His_6_-tags, were eluted with 20 ml of elution buffer (50 mM HEPES pH 7.4, 500 mM NaCl, 400 mM imidazole, 1 mM TCEP). Then, the portion of His_6_-tagged TEV protease (final concentration of 0.1 mg/ml) was added to the protein samples. The cleavage of the His_6_-tags from *At*SPDS1 and *At*SPDS2 was carried out in parallel to the overnight dialysis at 4°C against the buffer containing: 50 mM HEPES pH 8.0, 500 mM NaCl, 1 mM TCEP. Then, the samples were applied on the HisTrap HP resin in order to remove cleaved His_6_-tag and His_6_-tagged TEV protease. *At*SPDS1 and *At*SPDS2 were then concentrated with Amicon concentrators (Millipore) and applied on the HiLoad Superdex 200 16/60 column (GE Healthcare) connected to the AKTA FPLC system (Amersham Biosciences). Size-exclusion chromatography buffer contained 50 mM HEPES pH 7.4, 100 mM KCl, 50 mM NaCl, 1 mM TCEP.

### Crystallization and Data Collection

Crystallization trials were carried out parallelly for two constructs (full-length and truncated) of both *At*SPDS isoforms by the sitting drop method. Crystallization conditions of the full-length apo *At*SPDS1 were as follows: 16 mg/ml protein concentration, 0.2 M ammonium sulfate, 0.1 M BIS-TRIS at pH 5.5, 25% polyethylene glycol (PEG) 3350. Crystals were cryoprotected before diffraction experiment by 25% glycerol. Truncated apo *At*SPDS2 crystallized from 19 mg/ml in 76th conditions of the MORPHEUS screen (Gorrec, 2009). There was no necessity to use any cryoprotectant before freezing *At*SPDS2 crystals. The complex of *At*SPDS1 with dc-SAM and CHA was obtained by mixing truncated *At*SPDS1 at 23 mg/ml with ligands (10 mM final concentration of each ligand). Crystals were obtained by streak seeding in conditions containing 0.18 M ammonium sulfate, 0.09 M BIS-TRIS at pH 5.5, and 22% PEG 3350. 25% MPD was used as a cryoprotectant. For clarity, the complex of *At*SPDS1 with dc-SAM and CHA is further referred to as *At*SPDS1-CHA. The concentration of protein samples was determined spectrophotometrically at 280 nm using theoretical molar extinction coefficients calculated in *ProtParam* (Gasteiger et al., 2005).

The diffraction data were collected at the SER-CAT 22-BM beamline at the Advanced Photon Source (APS), Argonne National Laboratory, United States. The diffraction data were processed with *XDS* (Kabsch, 2010). Since the diffraction of all crystals demonstrated significant anisotropy, scaling of the data was performed with *STARANISO*^1^. The anisotropic cut-off surface for *At*SPDS1 data has been determined from 1.80 Å (best diffraction limits) to 3.14 Å (worst diffraction limits). In the case of *At*SPDS2, the anisotropic diffraction limits were between 2.0 and 2.77 Å. For the *At*SPDS1-CHA complex, the diffraction limits were between 1.80 and 2.70 Å. Table 1 provides detailed statistics for spherical and anisotropic truncation. After anisotropic truncation of the data, the electron density maps of all refined structures were significantly improved. Coordinates and structure factors were deposited in the PDB under the accession numbers 6o63 (*At*SPDS1), 6o64 (*At*SPDS2), and 6o65 (*At*SPDS1-CHA).

**Table 1:** Data collection and refinement statistics.

Structure	*At*SPDS1	*At*SPDS2	*At*SPDS1-CHA
**Data collection**			
Beamline	22-BM	22-BM	22-BM
Wavelength (Å)	1.00	1.00	1.00
Temperature (K)	100	100	100
Oscillation range (°)	0.25	0.25	0.25
Space group	*P*2_1_	*P*2_1_	*P*2_1_
Unit cell parameters (Å,°)	89.5, 76.3, 90.2 β = 104.7	75.6, 162.7, 97.5 β = 100.2	89.2, 107.6, 142.4 β = 95.3
Resolution^1^ (Å)	44.6–1.8^1a^ (1.86–1.8)	46.0–2.0^1b^ (2.07–2.0)	47.6–1.8^1c^ (1.85–1.8)
Reflections collected/unique	396350/95958 (19131/4794)	534541/125268 (26656/6263)	667473/200872 (34955/10041)
Completeness (%)			
Spherical	87.9 (45.6)	80.2 (42.8)	80.6 (44.0)
Ellipsoidal	95.4 (78.5)	97.1 (97.1)	93.4 (95.5)
Multiplicity	4.1 (4.0)	4.3 (4.3)	3.3 (3.5)
*R*_merge_ (%)	5.5 (54.7)	5.6 (55.7)	8.9 (41.3)
<*I*/σ(*I)*>	14.1 (2.5)	16.5 (2.5)	8.2 (2.7)
*CC_1/2_* (%)	99.9 (78.7)	99.9 (84.4)	99.7 (83.3)
**Refinement**			
*R_free_* reflections	1083	1234	1621
No. of atoms (non-H)			
Protein	8875	17520	18075
Ligands	62	134	290
Solvent	986	936	2103
*R*_work_/*R*_free_ (%)	16.5/22.2	17.6/23.1	21.9/26.1
Mean ADP^2^ (Å^2^)	25	35	18
RMSD^3^ from ideal geometry			
Bond lengths (Å)	0.013	0.014	0.016
Bond angles (^o^)	1.80	1.86	1.98
Ramachandran statistics (%)			
Favored	97	95	96
Allowed	3	5	4
Outliers	0	0	0
PDB code	6o63	6o64	6o65


*^1^Best anisotropic diffraction limit cut-off. ^*1a*^Worst diffraction limit after cut-off is 3.14 Å. ^*1b*^Worst diffraction limit after cut-off is 2.77 Å. ^*1c*^Worst diffraction limit after cut-off is 2.70 Å. ^*2*^ADP, atomic displacement parameter (B factor). ^*3*^RMSD, root-mean-square deviation. Values in parentheses refer to the highest resolution shell.*

### Structure Determination and Refinement

The previously deposited structure *At*SPDS1 (PDB ID: 1xj5, Center for Eukaryotic Structural Genomics, unpublished structure) was used as an initial model for the phase determination of our *At*SPDS1 structure. The model was then taken for the subsequent steps of manual and automatic refinement with *Coot* (Emsley et al., 2010) and *Refmac* (Murshudov et al., 2011). *TLS* parameters (Winn et al., 2003) were applied at the later stages of the structure refinement. In the case of *At*SPDS2 and *At*SPDS1-CHA structures, the coordinates of the dimer from the refined structure of *At*SPDS1 were used as a search model in *Phaser* (McCoy et al., 2007). The refinement was analogical to that of *At*SPDS1. *R*_work_, *R*_free_ factors (Brunger, 1992) and geometric parameters were controlled during refinement. The quality of refined structures was investigated in *PROCHECK* (Laskowski et al., 1993) and *MolProbity* (Chen et al., 2010). The final refinement statistics are given in Table 1. Geometrical restraints for CHA were generated in *eLBOW* (Moriarty et al., 2009).

### Small-Angle X-Ray Scattering Measurement

SAXS data were collected from the samples of the full-length *At*SPDS1 and the truncated construct of *At*SPDS2 at 7 and 4.5 mg/ml, respectively. The experiments were carried out at the BioCAT 18-ID beamline (Fischetti et al., 2004) at APS. The sample was applied to the WTC-015S5 column (Wyatt Technologies) coupled to the Infinity II HPLC (Agilent Technologies) system on the in-line size exclusion chromatography (SEC-SAXS) setup. After the column, the sample was analyzed with the Agilent UV detector, a Multi-Angle Light Scattering (MALS) detector and a Dynamic Light Scattering (DLS) detector (DAWN Helios II, Wyatt Technologies), and an RI detector (Optilab T-rEX, Wyatt). Then, it was sent to the SAXS flow cell, a 1.5 mm quartz capillary. The scattering intensity was collected with the exposure 0.5 and 2-s intervals at 1.03 Å wavelength at room temperature on a Pilatus3 1M detector (Dectris). The sample-to-detector distance was 3.5 m and the collected q-range was 0.004–0.4 Å^-1^. *BioXTAS* *RAW* 1.5.1 (Hopkins et al., 2017) was used for data reduction and analysis. To increase the signal-to-noise ratio several frames from to the elution peak of the chromatogram were averaged. The subtraction of the buffer signal from the sample scattering was done on the averaged frames directly proximal the sample peak. The *Rg* value calculated from the Guinier and distance distribution analysis were 29.4 and 29.6 Å for *At*SPDS1 and *At*SPDS2, respectively. The calculated maximum dimensions of the particles (*D*_max_) for *At*SPDS1 and *At*SPDS2 were 100 and 103 Å. The further calculation was performed with the *qRg* limits for 0.28–1.30 for *At*SPDS1 and 0.26–1.30 for *At*SPDS2. *DAMMIF* (Franke and Svergun, 2009), *DAMAVER* (Volkov and Svergun, 2003), *DAMMIN* (Svergun, 1999), and *DAMFILT* were consecutively used for the calculation of the *ab initio* envelopes, averaging, refinement and filtration. Twofold symmetry restraints were used for the envelope calculations. SAXS envelopes were superposed with the crystallographic dimers in *SUPCOMB*.

### Other Software Used

Molecular illustrations were made in UCSF *Chimera* (Pettersen et al., 2004). The sequence conservation scores were determined with *ConSurf* (Ashkenazy et al., 2016). The electrostatic potentials were calculated in *PDB2PQR* and *APBS* (Baker et al., 2001; Dolinsky et al., 2004). Polder omit maps were calculated in *Phenix* (Liebschner et al., 2017).

## Results and Discussion

### *At*SPDS1 and *At*SPDS2 Structures

**FIGURE 1:**
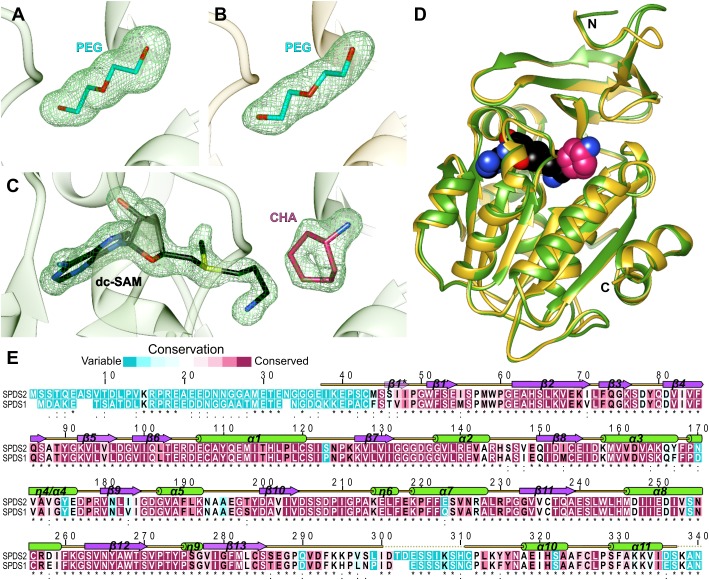
*At*SPDS structure. **(A)** PEG molecule bound in the chain B of *At*SPDS1 structure, **(B**) PEG molecule bound in the chain G of *At*SPDS2 structure, and **(C)** dc-SAM and CHA bound in the chain A of *At*SPDS1-CHA structure; green mesh represents omit maps (contoured at 4σ) calculated in *Phenix* (Liebschner et al., 2017). **(D)** Superposition of *At*SPDS1-CHA (chain A, green) and *At*SPDS2 (chain G, yellow) with the schematic depiction of the active site (dc-SAM is shown in black and CHA, which depicts the location of the polyamine groove, is shown in pink). **(E)** Sequence alignment of *At*SPDS2 (top) and *At*SPDS1 (bottom); residues are color-coded by the conservation score based on the results from *ConSurf* (Ashkenazy et al., 2016); the alignment of the flowering plant SPDS sequences derived from the phylogenetic analysis described in Sekula and Dauter (2018) was used in the calculations; the secondary structure elements are shown above the alignment: helices (green cylinders), sheets (violet arrows), and coil regions (yellow lines); regions that were disordered in the structures are marked with dotted lines; the provided sequence positions refer to the *At*SPDS2 sequence.

The structures of apo *At*SPDS1 and *At*SPDS2 present the PEG molecules (absorbed from the crystallization solution) bound within the active site (Figure 1A,B). The conformation of this linear ligand mimics PUT, providing the information about the substrate binding mode inside the catalytic pocket. The third determined structure is the crystal complex of *At*SPDS1 with two bound compounds, dc-SAM and CHA (Figure 1C), which precisely shows the binding mode of the cofactor and the inhibitor of SPDS.

*At*SPDS1 and *At*SPDS2 share the fold of polyamine biosynthesis proteins with the characteristic two-domain topology (Figure 1D). The N-terminal domain is smaller (about 100 residues). It is built of six β-strands that fold into two β-sheets, the two-stranded β-hairpin and the four-stranded antiparallel β-sheet. Additional β strand (Ser46-Ile48) is formed only in chain H of *At*SPDS2 (one of the eight chains in the asymmetric unit) creating three-stranded β-sheet at the N-terminus. In other chains of *At*SPDS2 structure and all chains of *At*SPDS1, the N-terminus is either more disordered or curved in a way that no additional β-strand is formed. The C-terminal domain has a Rossmann fold–like topology with a core β-sheet built of seven strands (five parallel and two antiparallel strands) that is buried between two helical bundles. The active site of *At*SPDS is formed between the N-terminal and C-terminal domains (Figure 1D).

The overall conformation of *At*SPDS1 and *At*SPDS2, and the distribution of the secondary structure elements are almost identical in these two isoforms (Figure 1D). Single chains of *At*SPDS1 and *At*SPDS2 superposed to each other present about 0.5 Å root-mean-square deviation. Also, the overall sequence conservation of plant SPDSs (Figure 1E) is high with many conserved regions that determine common characteristics of SPDSs in the plant kingdom. Only three regions show significantly lower conservation, and these are the N-terminus (about 45 residues), the region around the disordered loop between β13 and α10, and the C-terminal part (Figure 1E).

*At*SPDS1 and *At*SPDS2 share 83% sequence identity, however, when the highly variable and very flexible N-terminal part (up to Met45 of *At*SPDS2) is excluded from the alignment, the sequence identity is almost 90%. *At*SPDS1 is six-residues shorter than *At*SPDS2 and for the clarity of further analysis, all sequence positions that are identical in both *At*SPDSs are denoted with double numbering, e.g., Asp201/205 indicates aspartic acid in position 201 in *At*SPDS1 and 205 in *At*SPDS2. Four sequence gaps of *At*SPDS1 are in the disordered N-terminus. Two of the missing residues are placed in the long loop, which connects β13 and α10 (Figure 1E). Recently solved structure of another plant APT, TSPS from *M*. *truncatula* (*Mt*TSPS) (Sekula and Dauter, 2018), shows higher order in this region, which in *Mt*TSPS clearly folds into an additional helix.

### Biological Assembly of *At*SPDS1 and *At*SPDS2

**FIGURE 2:**
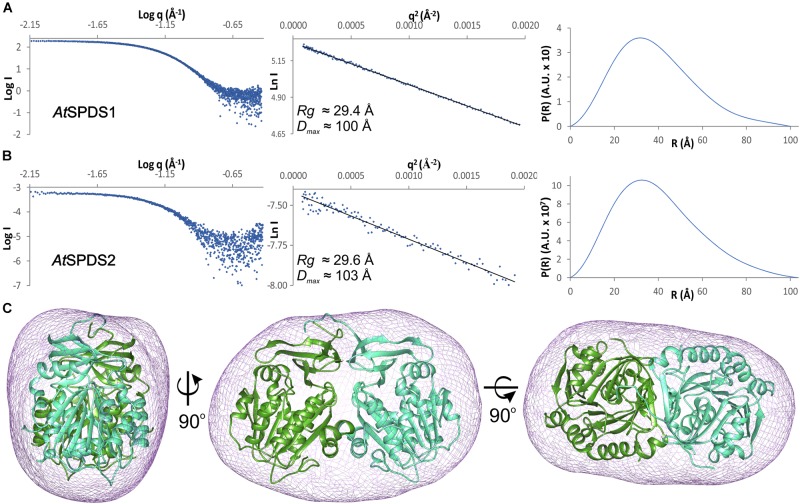
SAXS results. SAXS data for **(A)** *At*SPDS1 and **(B)** *At*SPDS2; charts present: the experimental SAXS curve (left), the Guinier plot of the scattering curve with the best fit shown as a black line (center), and the pair-distance distribution function for SAXS data (right). **(C)** *Ab* *initio* averaged SAXS envelope of *At*SPDS1 (violet mesh) superposed with the crystallographic *At*SPDS1 dimer (chains A and B shown in dark green and light green, respectively).

Both *A. thaliana* SPDS isoforms are dimers in solution. The estimated molecular weight of the full-length *At*SPDS1 calculated from the SAXS results (Figure 2A) is 74.5 kDa, which almost ideally matches the theoretical dimer mass (73 kDa). In the case of the truncated construct of *At*SPDS2, the SAXS results (Figure 2B) also matched the weight of the dimer (67.8 kDa in comparison to the theoretical value of 66.8 kDa). Also, the calculated *ab initio* envelope of *At*SPDS1 clearly corresponds to the *At*SPDS1 dimer in the crystal lattice (Figure 2C). The SAXS data of *At*SPDS2 presented significantly lower signal-to-noise ratio and the calculated *At*SPDS2 envelope (not shown) was worse in comparison to the envelope of *At*SPDS1, although it resembled the *At*SPDS2 crystallographic dimer in terms of its size. Slightly worse SAXS results for *At*SPDS2 can be explained by a lower concentration of the protein sample used for the analysis.

All crystal structures are solved in the monoclinic system with *P*2_1_ space group but in different crystal forms (Table 1). The asymmetric unit of apo *At*SPDS1 contains two dimers (A-B and C-D), while the complex of *At*SPDS1-CHA and the apo *At*SPDS2 both present four dimers (A-B, C-D, E-F, and G-H) in the asymmetric unit. The dimers of all structures are very similar. The dimer interface of *At*SPDS1 and *At*SPDS2 involves about 50 surface residues, that is about 15% of the subunit surface. It is created by the interactions of both domains (Figure 2C). In the case of the N-terminal domain, residues from the strand β2 and the loops connecting β4-β5 and β6-α1 have a major contribution to the dimer formation. In the C-terminal domains, interface residues are from the loop β11-α8, strand β12, and the two helices, α10 and α11. In the apo *At*SPDS1 crystal structure, dimers A-B and C-D (symmetry-related dimer) form an additional extensive buried surface between N-terminal domains of subunits A and C. This covers about 8% of the subunit surface and involves 12 hydrogen bonds which is recognized through PISA server (Krissinel and Henrick, 2007) as the interface important for oligomerization. No similar tight interdimeric interactions are observed in the other presented structures. Additionally, the SAXS results showing dimers of both *At*SPDS isoforms suggest that these additional interactions are rather the consequence of tight crystal packing of *At*SPDS1. On the other hand, it has been shown that plant APTs may form multiprotein complexes *in vivo* (Panicot et al., 2002) and the above mentioned interface may be involved in their formation. This somewhat similar tight interaction is actually responsible for the formation of *Mt*TSPS tetramer in the crystal and in solution (Sekula and Dauter, 2018), where the N-terminal β-hairpins form eight-stranded β-barrel.

Most of the characterized APTs are dimers, analogously to *At*SPDS1 and *At*SPDS2. These include, e.g., *E. coli* SPDS (*Ec*SPDS, PDB ID: 3o4f) (Zhou et al., 2010) or human SPDS (*Hs*SPDS, PDB ID: 2o05) (Wu et al., 2007). The exceptions are the tetrameric SPDSs, like *Thermotoga maritima* SPDS (*Tm*SPDS, PDB ID: 1inl) (Korolev et al., 2002), *Bacillus subtilis* SPDS (*Bs*SPDS, PDB ID: 1iy9), or *Helicobacter pylori* SPDS (*Hp*SPDS, PDB ID: 2cmg) (Lu et al., 2007). In some conditions, the latter protein can form either dimers or tetramers (Lu et al., 2007). The biological assembly of the tetrameric SPDSs more resembles the tetramer of *Mt*TSPS with the β-barrel formed by the N-terminal β-hairpins of the four subdomains (Sekula and Dauter, 2018) rather than the formation between two dimers observed in the apo crystal structure of *At*SPDS1.

### The Active Site of *At*SPDS

The large catalytic cavity on the interface between the N-terminal and C-terminal domains is the region of *At*SPDS with the highest negative charge. This feature can be easily explained by the necessity of *At*SPDS to attract dc-SAM and PUT, both presenting the cationic character. The active site is composed of two main compartments, the dc-SAM binding site and the polyamine binding grove. Cofactor binding site is placed closer to the C-terminal domain, while the substrate site is rather buried in the N-terminal domain.

**FIGURE 3:**
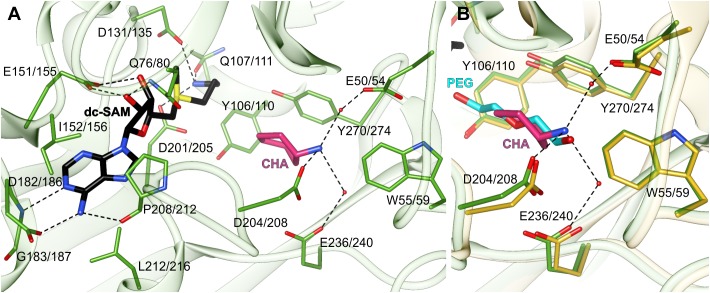
The active site of *At*SPDS. **(A)** The binding mode of dc-SAM (black) and CHA (pink) shown in the chain A of *At*SPDS1-CHA structure (green); dashed lines indicate hydrogen bonds; residues are numbered accordingly to the sequence positions of *At*SPDS1 and *At*SPDS2, respectively. **(B)** Comparison of the binding mode of CHA (pink) in chain A of *At*SPDS1 structure (green) with PEG molecule (cyan) bound in chain G of *At*SPDS2 structure (yellow).

The dc-SAM binding site stretches alongside the glycine-rich region between β7 and α2 (residues Gly128/132-Gly133/137). Its boundaries are marked by Asp182/186 from one side and Gln107/111 from the other side (Figure 3A). Glu151/155, which is responsible for the H-bonding interactions with ribosyl moiety of dc-SAM, virtually divides the dc-SAM binding site into two compartments that accommodate adenosine and aminopropyl moieties, respectively (Figure 3A). The bulky compartment which facilitates adenosine moiety of the cofactor is covered by the very flexible region that comprises η6 together with the flanking loops (see below). The adenosine moiety of the dc-SAM is stacked between Leu212/216 and Ile152/156 and it forms three hydrogen bonds with the surrounding residues (Figure 3A). Two of these H-bonds are created by the N^6^ amine with the carbonyl oxygen atom of Pro208/212 and the OD1 oxygen atom of Asp182/186. The third hydrogen bond is created between the N^1^ of dc-SAM and the backbone amide of Gly183/187. The aminopropyl binding site is significantly smaller. The niche is formed by polar residues, Asp131/135, Asp201/205, and Gln107/111 that create three hydrogen bonds with terminal amine of the cofactor’s aminopropyl moiety (Figure 3A). Asp201/205 is placed in a way to reach and to deprotonate the amine group of PUT before the reaction. Deprotonated PUT can perform the nucleophilic attack on the carbon atom of the dc-SAM aminopropyl moiety. A very similar hydrogen bonds network between dc-SAM and surrounding residues are present in other SPDSs, like human *Hs*SPDS (PDB ID: 2o0l) (Wu et al., 2007). Also, the analogical residues that correspond to Ile152/156 and Leu212/216 are responsible for the stacking of the adenine base of dc-SAM. Plant SPMSs present almost identical highly conserved primary structure of the dc-SAM binding site as *At*SPDS. On the other hand, *Mt*TSPS (PDB ID 6bq2) (Sekula and Dauter, 2018) shows some differences inside the cofactor binding site. The first difference is the residue that corresponds to Glu151/155 of *At*SPDS and binds the ribose moiety of dc-SAM. In *Mt*TSPS, and in other plant TSPSs, it is Asp129. The other difference concerns Gln107/111, which in *Mt*TSPS is replaced with His. Also, the adenine base is differently stabilized by the apolar residues. The residue corresponding to Leu212/216 is the same, but the difference concerns the interactions from the other side of the plane of the adenine base. In *Mt*TSPS the function of Leu212/216 takes Leu179, the residue in a position corresponding to Ser202/206 of *At*SPDS.

The polyamine binding site is an elongated tunnel-shaped cavity that stretches deep down the N-terminal domain, from Asp201/205 to Trp55/59. Trp55/59, which is a part of the N-terminal β-hairpin, limits the length of the cavity and shapes its bottom wall. The key residues in this part of the active site are easily recognized in the structure of *At*SPDS1-CHA, where the polyamine groove is occupied by the inhibitor. CHA is bound close to two perpendicularly situated Tyr residues, Tyr106/110 and Tyr270/274, and its amine group crates three hydrogen bonds with acidic residues at the bottom of the polyamine grove (Figure 3A). These are the direct hydrogen bond with Asp208, and the two water-mediated H-bonds with Glu236/240 and Glu50/54. The position of the amine group of CHA overlaps with the PEG molecule that is bound in the apo structures (Figure 3B). The bound PEG molecule stretches along the polyamine groove, resembling PUT bound in other SPDS structures. Therefore, it is highly probable that PUT molecule in *At*SPDS creates a very similar hydrogen bond network to CHA at the bottom of the pocket. The aliphatic portion of PUT is most likely stabilized by the interactions with two perpendicularly positioned aromatic side chains of Tyr106/110 and Tyr270/274 so that the other end of PUT can be pointed close to the cofactor, where it can be deprotonated by Asp201/205 and initialize the transfer of the aminopropyl moiety.

The ligands in the polyamine grove of *At*SPDS do not reach as deep to the bottom of the active site as bound SPD in *Mt*TSPS (PDB ID: 6bq7) (Sekula and Dauter, 2018). The reason that the amine group of SPD in *Mt*TSPS is placed deeper inside the cleft where it creates a direct hydrogen bond with Glu30 (Glu50/54 of *At*SPDS) is the necessity of *Mt*TSPS to accommodate longer substrate in order to synthesize TSP. This difference in the polyamine binding mode between SPDS and TSPS is caused by several features of the N-terminal β-hairpin that distinguish these two plant APTs and determine their substrate discriminatory features (Sekula and Dauter, 2018). Additionally, Asp181 of *Mt*TSPS (Asp204/208 in *At*SPDS), the residue which is placed in the loop close to the η6 and in *At*SPDS H-bonds the amine group of the substrate, in *Mt*TSPS is rotated outside the polyamine grove and interacts with Gln214 instead of the substrate. In comparison to plant SPMSs, SPDSs lack the additional insert (about 20 residues) in the N-terminal β-hairpin (Sekula and Dauter, 2018) which presumably differentiates the shape of the polyamine groove between plant SPDSs and SPMSs, and therefore determines their different specificity.

**FIGURE 4:**
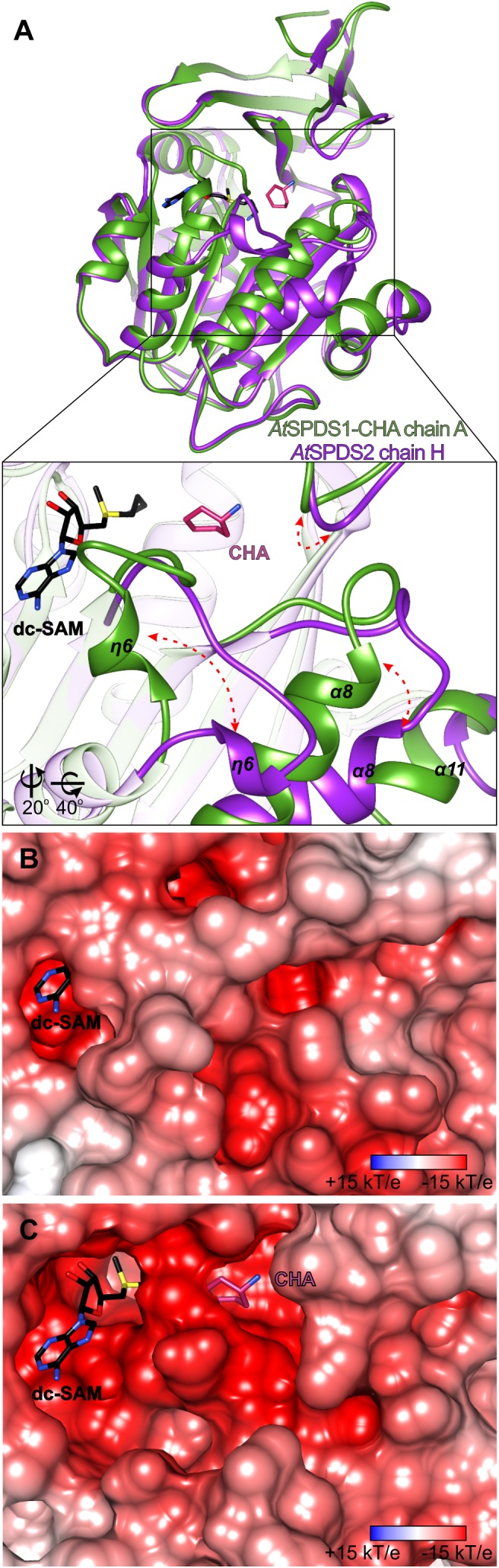
Structural rearrangements inside the active site of *At*SPDS. **(A)** Superposition of chain A of *At*SPDS1-CHA structure (green) and chain H of *At*SPDS2 (violet), which represents “closed” and “open” conformations of *At*SPDS, respectively; rectangle indicates the region zoomed-in in the bottom panel; red arrows indicate major movements that occur upon ligand binding; note that the view in the bottom panel has been changed by the application of appropriate rotation (bottom left corner). Charge distribution mapped on the surface representation around the active site **(B)** in the closed conformation (chain A of *At*SPDS1) and **(C)** in the open conformation (chain H of *At*SPDS2); dc-SAM (black) and CHA (violet) are superposed from the *At*SPDS1-CHA to indicate the location of the cofactor and the substrate binding sites. Orientation in the panels **(B,C)** is identical to the bottom view of the panel **(A)**. The calculation of the electrostatic potential assuming pH 7.3 was made in *PDB2PQR* and *APBS* (Baker et al., 2001; Dolinsky et al., 2004).

The binding mode of CHA by *At*SPDS1 is very similar to the binding mode of cyclic and aromatic CHA analogs in *Trypanosoma cruzi* SPDS (PDB ID 4yuw) (Amano et al., 2015) and *Plasmodium falciparum* SPDS (*Pf*SPDS, PDB IDs: 4bp3, 4uoe, and 2pt9) (Dufe et al., 2007; Sprenger et al., 2015). In the case of *Pf*SPDS, the authors observed that the protein requires the stabilization of the flexible region with η6 together with the flanking loops to actually bind the inhibitor (Sprenger et al., 2015). A similar observation was made with *Hs*SPDS where PUT was bound in the active site only when the cofactor was present (Wu et al., 2007). In the case of *At*SPDS1 and *At*SPDS2, we have also observed a somewhat similar situation. We have tried to soak the apo *At*SPDS crystals with CHA or to cocrystallize *At*SPDS with CHA alone, but these attempts were unsuccessful. On the other hand, both apo structures, even though there was no ligand in the dc-SAM binding site, presented bound PEG fragment inside the polyamine groove. It is true for all, but one chains of the two apo structures of *At*SPDS. All chains present the conformation very similar to that shown in Figure 1D, which from now on is referred to as a closed state. One subunit of the *At*SPDS2 (chain H) presents the state, where no ligand is bound in the active site in neither the cofactor binding compartment nor in the polyamine groove. The conformation of this chain stands out from the others and presents the possible open conformation of the active site (Figure 4A).

### Conformational Movement of *At*SPDS

Similarly to the other SPDS enzymes, also *At*SPDS stabilizes upon ligand binding. This feature is even more emphasized when temperature factors of the two very similar (in terms of resolution) structures are compared – apo *At*SPDS1 and *At*SPDS1-CHA. Apo structure, where no cofactor is bound in the binding site, has an average B factor significantly higher than the complexed structure. Comparison of the chain H of *At*SPDS2 in the open conformation with chain A of the *At*SPDS1-CHA complex (Figure 4A) shows that the *At*SPDS adopts two significantly different conformations. Globally, the main differences between the open (without ligands) and closed (with bound ligands) states concern the following regions (Figure 4A): η6 together with the flanking loops (residues 203/207-214/218), α8 with the preceding loop (residues 235/239-252/256), the loop of the N-terminal β-hairpin (residues 51/55-57/61) and the C-terminus.

In the first-mentioned region, in the closed conformation, the helix η6 is almost perpendicular to the next helix α7 in a way that it entirely covers the cofactor binding site (Figure 4B). Moreover, the loop region is curved in a way that Asp204/208 can reach CHA (or PUT) to create hydrogen bond with its amine group and to stabilize the substrate during the catalysis. In the second region in the closed state, helix α8 is positioned parallelly to the β13 strand of the core β-sheet. The C-terminus is quite well structured and visible in the electron density map up to Ser334/337. Also, the loop of the N-terminal β-hairpin is positioned close to the active site.

In the open conformation, the biggest conformational difference concerns the region with η6. The preceding loop uncoils and η6 is now moved toward α8, over 10 Å away from the position in the closed form. Therefore, the negatively charged active site is uncovered and ready to incorporate cofactor and substrate (Figure 4C). This opening of the active site is possible due to the concerted movement of the other parts of the protein as well. The beginning of α8 helix is shifted almost 6 Å away in comparison to the closed conformation. This shift has an impact not only on the conformation of the preceding loop but also on the C-terminal helix α11, which is also shifted, and it becomes more disordered. Also, the loop of the N-terminal β-hairpin slightly moves outside the pocket (Figure 4A) in the open state, which has a serious consequence for the substrate/inhibitor binding (see below). It is worth noting that the opened conformation of the chain H of *At*SPDS2 was possible to capture only due to the crystal packing, where the residues created additional H-bonds with a symmetry-related unit in the crystal lattice.

**FIGURE 5:**
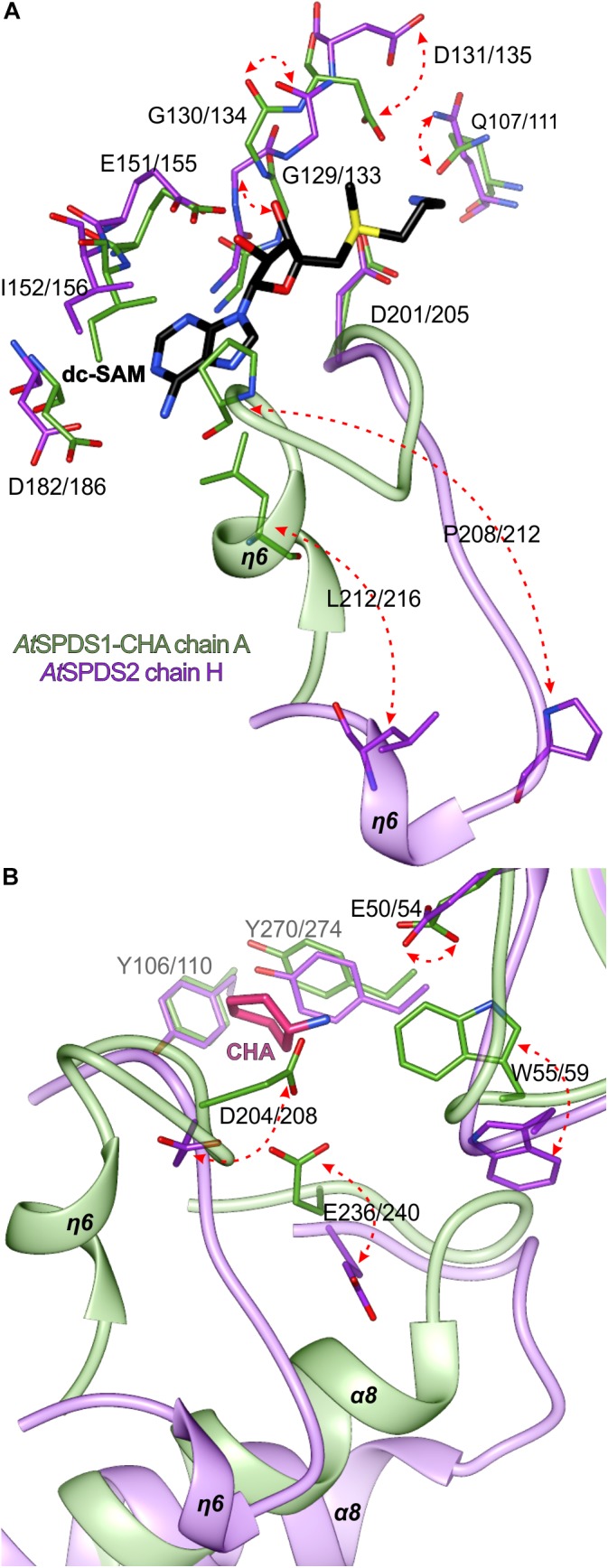
Structural changes of *At*SPDS upon ligand binding. The detailed conformational differences between the closed (chain A of *At*SPDS1-CHA structure, green) and open (chain H of *At*SPDS2 structure, violet) conformations around the cofactor binding site **(A)** and the polyamine groove **(B)**; red arrows indicate major movements; note that the bound dc-SAM (black) and CHA (pink) are bound only in the chain A of *At*SPDS1-CHA; residues are numbered accordingly to the sequence positions of *At*SPDS1 and *At*SPDS2, respectively.

The major transition between open and closed state of *At*SPDS around the cofactor binding site (Figure 5A) involves Leu212/216 and Pro208/212, residues from the η6 region that are crucial for the dc-SAM stabilization. Additionally, residues in the glycine-rich region change their position to facilitate dc-SAM. Gly129/133 and Gly130/134 alter their conformation, which in consequence moves the Asp131/135 that is now poised to form a hydrogen bond with the amine group of dc-SAM. Simultaneously, the side chain of Gln107/111 rotates to complement the hydrogen-bonding network with dc-SAM.

Most likely, when the η6 is positioned in the closed conformation after the dc-SAM incorporation, the polyamine grove is adapted for substrate/inhibitor binding. Asp204/208 is rotated to form a hydrogen bond with an amine group of bound ligand inside the polyamine grove (Figure 5B). Also, together with the movement of α8, Glu236/240 is moved inside the active site. The movement of α8 probably has also the impact on the conformation of Trp55/59, which is pushed inside the cleft to shape the bottom wall of the polyamine grove. Also, when the ligand is bound, the side chain of Glu50/54 rotates to form a water-mediated H-bond with the ligand in the polyamine grove.

The fact that SPDS enzymes require the cofactor to be bound first in the dc-SAM binding site can be explained by the necessity of the gate region with η6 to be stabilized in the closed conformation. This helps to preserve the position of Asp204/208 and Glu236/240 in a way that they can easily create H-bonds with bound substrate or inhibitor. Most likely, in the absence of dc-SAM inside the active site the gate region is too unstable, therefore the ligand inside the polyamine grove cannot be sufficiently stabilized. The search across the PDB shows that the region with η6 in most of the APTs is disordered without the ligand bound inside the dc-SAM binding site. *E. coli* SPDS (PDB ID 3o4f) (Zhou et al., 2010) is another example, where some chains were captured in open conformation, similarly to chain H of *At*SPDS2. On the other hand, such high instability of the η6 without ligands was not observed in *Mt*TSPS (Sekula and Dauter, 2018). Probably, *Mt*TSPS presents a different mechanism to open the catalytic cleft, where the active site may be opened through a relative movement of C-terminal domains with respect to the N-terminal intersubunit β-barrel.

## Conclusion

In this work, we have presented the crystal structures of two isoforms of SPDS from *A. thaliana*, *At*SPDS1 and *At*SPDS2, and compared the unbound and the bound conformations of these enzymes. The structures show the binding mode of dc-SAM, a universal cofactor of APTs and the donor of the aminopropyl moiety. The *At*SPDS1-CHA structure gave insights into the inhibition of the plant SPDSs by CHA. This competitive inhibitor binds inside the polyamine groove of the active site creating three hydrogen bonds at the bottom of the pocket, analogical to these created by the bound substrate. Inside the polyamine grove, the inhibitor is also stabilized by the hydrophobic interactions with two perpendicularly situated Tyr residues, which also stabilize PUT. The crystallographic snapshots show in detail the structural rearrangements around the active site of *At*SPDS that are required to facilitate both, the cofactor and the substrate/inhibitor. The protein undergoes concerted movement of the three major parts (i) close to the glycine-rich region where aminopropyl moiety of dc-SAM is bound, (ii) the very flexible gate region with η6, where residues interact with the adenine moiety of dc-SAM and the bound polyamine/inhibitor, and (iii) the N-terminal β-hairpin, that limits the PUT binding grove at the bottom.

## Data Availability

The datasets generated for this study can be found in Protein Data Bank, under the accession codes 6o63 (*At*SPDS1), 6o64 (*At*SPDS2), and 6o65 (*At*SPDS1-CHA).

## Author Contributions

BS planned and performed the experiments, analyzed the results, and wrote the manuscript. ZD analyzed the results and supervised the work.

## Conflict of Interest Statement

The authors declare that the research was conducted in the absence of any commercial or financial relationships that could be construed as a potential conflict of interest.

## Footnotes

1 http://staraniso.globalphasing.org/cgi-bin/staraniso.cgi

## Abbreviations

APT: aminopropyltransferase
CHA: cyclohexylamine
dc-SAM: decarboxylated S-adenosylmethionine
PUT: putrescine
SPD: spermidine
SPDS: spermidine synthase
SPMS: spermine synthase
TSPS: thermospermine synthase

## References

[B1] Alcázar R. Altabella T. Marco F. Bortolotti C. Reymond M. Koncz C. (2010). Polyamines: molecules with regulatory functions in plant abiotic stress tolerance. *Planta* 231 1237–1249. 10.1007/s00425-010-1130-0 20221631

[B2] Amano Y. Namatame I. Tateishi Y. Honboh K. Tanabe E. Niimi T. (2015). Structural insights into the novel inhibition mechanism of *Trypanosoma cruzi* spermidine synthase. *Acta Crystallogr. D Biol. Crystallogr.* 71 1879–1889. 10.1107/S1399004715013048 26327378

[B3] Ashkenazy H. Abadi S. Martz E. Chay O. Mayrose I. Pupko T. (2016). ConSurf 2016: an improved methodology to estimate and visualize evolutionary conservation in macromolecules. *Nucleic Acids Res.* 44 W344–W350. 10.1093/nar/gkw408 27166375 PMC4987940

[B4] Baker N. A. Sept D. Joseph S. Holst M. J. McCammon J. A. (2001). Electrostatics of nanosystems: application to microtubules and the ribosome. *Proc. Natl. Acad. Sci. U.S.A.* 98 10037–10041. 10.1073/pnas.181342398 11517324 PMC56910

[B5] Belda-Palazón B. Almendáriz C. Martí E. Carbonell J. Ferrando A. (2016). Relevance of the Axis Spermidine/eIF5A for plant growth and development. *Front. Plant Sci.* 7:245. 10.3389/fpls.2016.00245 26973686 PMC4773603

[B6] Belda-Palazon B. Ruiz L. Marti E. Tarraga S. Tiburcio A. F. Culianez F. (2012). Aminopropyltransferases involved in polyamine biosynthesis localize preferentially in the nucleus of plant cells. *PLoS One* 7:e46907. 10.1371/journal.pone.0046907 23056524 PMC3466176

[B7] Bouchereau A. Aziz A. Larher F. Martin-Tanguy J. (1999). Polyamines and environmental challenges: recent development. *Plant Sci.* 140 103–125. 10.1016/S0168-9452(98)00218-0

[B8] Brunger A. T. (1992). Free R value: a novel statistical quantity for assessing the accuracy of crystal structures. *Nature* 355 472–475. 10.1038/355472a0 18481394

[B9] Burhenne K. Kristensen B. K. Rasmussen S. K. (2003). A new class of N-hydroxycinnamoyltransferases. Purification, cloning, and expression of a barley agmatine coumaroyltransferase (EC 2.3.1.64). *J. Biol. Chem.* 278 13919–13927. 10.1074/jbc.M213041200 12582168

[B10] Chen V. B. Arendall W. B. Headd J. J. Keedy D. A. Immormino R. M. Kapral G. J. (2010). MolProbity: all-atom structure validation for macromolecular crystallography. *Acta Crystallogr. D Biol. Crystallogr.* 66 12–21. 10.1107/S0907444909042073 20057044 PMC2803126

[B11] Dolinsky T. J. Nielsen J. E. McCammon J. A. Baker N. A. (2004). PDB2PQR: an automated pipeline for the setup of poisson-boltzmann electrostatics calculations. *Nucleic Acids Res.* 32 W665–W667. 10.1093/nar/gkh381 15215472 PMC441519

[B12] Dufe V. T. Qiu W. Muller I. B. Hui R. Walter R. D. Al-Karadaghi S. (2007). Crystal structure of *Plasmodium falciparum* spermidine synthase in complex with the substrate decarboxylated S-adenosylmethionine and the potent inhibitors 4MCHA and AdoDATO. *J. Mol. Biol.* 373 167–177. 10.1016/j.jmb.2007.07.053 17822713

[B13] Emsley P. Lohkamp B. Scott W. G. Cowtan K. (2010). Features and development of Coot. *Acta Crystallogr. D Biol. Crystallogr.* 66 486–501. 10.1107/S0907444910007493 20383002 PMC2852313

[B14] Fischetti R. Stepanov S. Rosenbaum G. Barrea R. Black E. Gore D. (2004). The BioCAT undulator beamline 18ID: a facility for biological non-crystalline diffraction and X-ray absorption spectroscopy at the advanced photon source. *J. Synchrotron Radiat.* 11 399–405. 10.1107/S0909049504016760 15310956

[B15] Franke D. Svergun D. I. (2009). DAMMIF, a program for rapid ab-initio shape determination in small-angle scattering. *J. Appl. Crystallogr.* 42 342–346. 10.1107/S0021889809000338 27630371 PMC5023043

[B16] Gallardo M. Gallardo M. E. Matilla A. J. de Rueda P. M. Sánchez-Calle I. M. (1994). Inhibition of polyamine synthesis by cyclohexylamine stimulates the ethylene pathway and accelerates the germination of Cicer arietinum seeds. *Physiol. Plant* 91 9–16. 10.1111/j.1399-3054.1994.tb00652.x

[B17] Gasteiger E. Hoogland C. Gattiker A. Duvaud S. E. Wilkins M. R. Appel R. D. (2005). “Protein Identification and Analysis Tools on the ExPASy Server,” in *The Proteomics Protocols Handbook*, ed. Walker J. M. (Totowa, NJ: Humana Press), 571–607. 10.1385/1-59259-890-0:571

[B18] Gill S. S. Tuteja N. (2010). Polyamines and abiotic stress tolerance in plants. *Plant Signal. Behav.* 5 26–33. 10.4161/psb.5.1.10291 20592804 PMC2835953

[B19] Gonzalez M. E. Marco F. Minguet E. G. Carrasco-Sorli P. Blazquez M. A. Carbonell J. (2011). Perturbation of spermine synthase gene expression and transcript profiling provide new insights on the role of the tetraamine spermine in Arabidopsis defense against *Pseudomonas viridiflava*. *Plant Physiol.* 156 2266–2277. 10.1104/pp.110.171413 21628628 PMC3149955

[B20] Gorrec F. (2009). The MORPHEUS protein crystallization screen. *J. Appl. Crystallogr.* 42 1035–1042. 10.1107/S0021889809042022 22477774 PMC3246824

[B21] Hanfrey C. Sommer S. Mayer M. J. Burtin D. Michael A. J. (2001). Arabidopsis polyamine biosynthesis: absence of ornithine decarboxylase and the mechanism of arginine decarboxylase activity. *Plant J.* 27 551–560. 10.1046/j.1365-313X.2001.01100.x 11576438

[B22] Hanzawa Y. Takahashi T. Michael A. J. Burtin D. Long D. Pineiro M. (2000). ACAULIS5, an Arabidopsis gene required for stem elongation, encodes a spermine synthase. *EMBO J.* 19 4248–4256. 10.1093/emboj/19.16.4248 10944107 PMC302034

[B23] Hashimoto T. Shoji T. Mihara T. Oguri H. Tamaki K. Suzuki K.-I. (1998a). Intraspecific variability of the tandem repeats in Nicotiana putrescine N-methyltransferases. *Plant Mol. Biol.* 37 25–37. 10.1023/a:1005961122814 9620262

[B24] Hashimoto T. Tamaki K. Suzuki K. Yamada Y. (1998b). Molecular cloning of plant spermidine synthases. *Plant Cell Physiol.* 39 73–79. 10.1093/oxfordjournals.pcp.a029291 9517003

[B25] Hopkins J. B. Gillilan R. E. Skou S. (2017). BioXTAS RAW: improvements to a free open-source program for small-angle X-ray scattering data reduction and analysis. *J. Appl. Crystallogr.* 50 1545–1553. 10.1107/S1600576717011438 29021737 PMC5627684

[B26] Hu L. Xiang L. Zhang L. Zhou X. Zou Z. Hu X. (2014). The photoprotective role of spermidine in tomato seedlings under salinity-alkalinity stress. *PLoS One* 9:e110855. 10.1371/journal.pone.0110855 25340351 PMC4207769

[B27] Igarashi K. Kashiwagi K. (2010). Modulation of cellular function by polyamines. *Int. J. Biochem. Cell Biol.* 42 39–51. 10.1016/j.biocel.2009.07.009 19643201

[B28] Jiménez-Bremont J. F. Marina M. Guerrero-González M. D. L. L. Rossi F. R. Sánchez-Rangel D. Rodríguez-Kessler M. (2014). Physiological and molecular implications of plant polyamine metabolism during biotic interactions. *Front. Plant Sci.* 5:95. 10.3389/fpls.2014.00095 24672533 PMC3957736

[B29] Kabsch W. (2010). Xds. *Acta Crystallogr. D Biol. Crystallogr.* 66 125–132. 10.1107/S0907444909047337 20124692 PMC2815665

[B30] Kamiab F. Talaie A. Khezri M. Javanshah A. (2014). Exogenous application of free polyamines enhance salt tolerance of pistachio (Pistacia vera L.) seedlings. *Plant Growth Regul.* 72 257–268. 10.1007/s10725-013-9857-9

[B31] Kasukabe Y. He L. Nada K. Misawa S. Ihara I. Tachibana S. (2004). Overexpression of spermidine synthase enhances tolerance to multiple environmental stresses and up-regulates the expression of various stress-regulated genes in transgenic Arabidopsis thaliana. *Plant Cell Physiol.* 45 712–722. 10.1093/pcp/pch083 15215506

[B32] Kim Y. Babnigg G. Jedrzejczak R. Eschenfeldt W. H. Li H. Maltseva N. (2011). High-throughput protein purification and quality assessment for crystallization. *Methods* 55 12–28. 10.1016/j.ymeth.2011.07.010 21907284 PMC3690762

[B33] Korolev S. Ikeguchi Y. Skarina T. Beasley S. Arrowsmith C. Edwards A. (2002). The crystal structure of spermidine synthase with a multisubstrate adduct inhibitor. *Nat. Struct. Biol.* 9 27–31. 10.1038/nsb737 11731804 PMC2792006

[B34] Krissinel E. Henrick K. (2007). Inference of macromolecular assemblies from crystalline state. *J. Mol. Biol.* 372 774–797. 10.1016/j.jmb.2007.05.022 17681537

[B35] Laskowski R. A. Macarthur M. W. Moss D. S. Thornton J. M. (1993). Procheck - a program to check the stereochemical quality of protein structures. *J. Appl. Crystallogr.* 26 283–291. 10.1107/S0021889892009944

[B36] Liebschner D. Afonine P. V. Moriarty N. W. Poon B. K. Sobolev O. V. Terwilliger T. C. (2017). Polder maps: improving OMIT maps by excluding bulk solvent. *Acta Crystallogr. D Struct. Biol.* 73 148–157. 10.1107/s2059798316018210 28177311 PMC5297918

[B37] Lu P. K. Tsai J. Y. Chien H. Y. Huang H. Chu C. H. Sun Y. J. (2007). Crystal structure of *Helicobacter* pylori spermidine synthase: a rossmann-like fold with a distinct active site. *Proteins* 67 743–754. 10.1002/prot.21315 17357156

[B38] McCoy A. J. Grosse-Kunstleve R. W. Adams P. D. Winn M. D. Storoni L. C. Read R. J. (2007). Phaser crystallographic software. *J. Appl. Crystallogr.* 40 658–674. 10.1107/s0021889807021206 19461840 PMC2483472

[B39] Michael A. J. (2017). Evolution of biosynthetic diversity. *Biochem. J.* 474 2277–2299. 10.1042/BCJ20160823 28655863

[B40] Minguet E. G. Vera-Sirera F. Marina A. Carbonell J. Blazquez M. A. (2008). Evolutionary diversification in polyamine biosynthesis. *Mol. Biol. Evol.* 25 2119–2128. 10.1093/molbev/msn161 18653732

[B41] Moriarty N. W. Grosse-Kunstleve R. W. Adams P. D. (2009). Electronic ligand builder and optimization workbench (eLBOW): a tool for ligand coordinate and restraint generation. *Acta Crystallogr. D Biol. Crystallog.* 65 1074–1080. 10.1107/S0907444909029436 19770504 PMC2748967

[B42] Mostofa M. G. Yoshida N. Fujita M. (2014). Spermidine pretreatment enhances heat tolerance in rice seedlings through modulating antioxidative and glyoxalase systems. *Plant Growth Regul.* 73 31–44. 10.1007/s10725-013-9865-9

[B43] Murshudov G. N. Skubak P. Lebedev A. A. Pannu N. S. Steiner R. A. Nicholls R. A. (2011). REFMAC5 for the refinement of macromolecular crystal structures. *Acta Crystallogr. D Biol. Crystallogr.* 67 355–367. 10.1107/S0907444911001314 21460454 PMC3069751

[B44] Panicot M. Minguet E. G. Ferrando A. Alcazar R. Blazquez M. A. Carbonell J. (2002). A polyamine metabolon involving aminopropyl transferase complexes in Arabidopsis. *Plant Cell* 14 2539–2551. 10.1105/tpc.004077 12368503 PMC151234

[B45] Pettersen E. F. Goddard T. D. Huang C. C. Couch G. S. Greenblatt D. M. Meng E. C. (2004). UCSF Chimera–a visualization system for exploratory research and analysis. *J. Comput. Chem.* 25 1605–1612. 10.1002/jcc.20084 15264254

[B46] Pottosin I. Shabala S. (2014). Polyamines control of cation transport across plant membranes: implications for ion homeostasis and abiotic stress signaling. *Front. Plant Sci.* 5:154. 10.3389/fpls.2014.00154 24795739 PMC4006063

[B47] Pottosin I. Velarde-Buendia A. M. Bose J. Fuglsang A. T. Shabala S. (2014). Polyamines cause plasma membrane depolarization, activate Ca2+-, and modulate H+-ATPase pump activity in pea roots. *J. Exp. Bot.* 65 2463–2472. 10.1093/jxb/eru133 24723394

[B48] Radhakrishnan R. Lee I. J. (2013). Spermine promotes acclimation to osmotic stress by modifying antioxidant, abscisic acid, and jasmonic acid signals in soybean. *J. Plant Growth Regul.* 32 22–30. 10.1007/s00344-012-9274-8

[B49] Sekula B. Dauter Z. (2018). Crystal structure of thermospermine synthase from *Medicago truncatula* and substrate discriminatory features of plant aminopropyltransferases. *Biochem. J.* 475 787–802. 10.1042/bcj20170900 29367265 PMC7983153

[B50] Sekula B. Dauter Z. (2019). Structural study of agmatine iminohydrolase from *Medicago truncatula*, the second enzyme of the agmatine route of putrescine biosynthesis in plants. *Front. Plant Sci.* 10:320. 10.3389/fpls.2019.00320 30984210 PMC6447857

[B51] Sekula B. Ruszkowski M. Dauter Z. (2018). Structural analysis of phosphoserine aminotransferase (Isoform 1) from arabidopsis thaliana– the enzyme involved in the phosphorylated pathway of serine biosynthesis. *Front. Plant Sci.* 9:876. 10.3389/fpls.2018.00876 30034403 PMC6043687

[B52] Sekula B. Ruszkowski M. Malinska M. Dauter Z. (2016). Structural investigations of N-carbamoylputrescine amidohydrolase from *Medicago truncatula*: insights into the ultimate step of putrescine biosynthesis in plants. *Front. Plant Sci.* 7:350. 10.3389/fpls.2016.00350 27066023 PMC4812014

[B53] Shao L. Majumdar R. Minocha S. C. (2012). Profiling the aminopropyltransferases in plants: their structure, expression and manipulation. *Amino Acids* 42 813–830. 10.1007/s00726-011-0998-8 21861167

[B54] Shirahata A. Morohohi T. Fukai M. Akatsu S. Samejima K. (1991). Putrescine or spermidine binding site of aminopropyltransferases and competitive inhibitors. *Biochem. Pharmacol.* 41 205–212. 10.1016/0006-2952(91)90478-N 1989632

[B55] Sprenger J. Svensson B. Halander J. Carey J. Persson L. Al-Karadaghi S. (2015). Three-dimensional structures of Plasmodium falciparum spermidine synthase with bound inhibitors suggest new strategies for drug design. *Acta Crystallogr. D Biol. Crystallogr.* 71 484–493. 10.1107/s1399004714027011 25760598 PMC4356361

[B56] Svergun D. I. (1999). Restoring low resolution structure of biological macromolecules from solution scattering using simulated annealing. *Biophys. J.* 76 2879–2886. 10.1016/S0006-3495(99)77443-6 10354416 PMC1300260

[B57] Tiburcio A. F. Altabella T. Bitrian M. Alcazar R. (2014). The roles of polyamines during the lifespan of plants: from development to stress. *Planta* 240 1–18. 10.1007/s00425-014-2055-9 24659098

[B58] Volkov V. V. Svergun D. I. (2003). Uniqueness of ab initio shape determination in small-angle scattering. *J. Appl. Crystallogr.* 36 860–864. 10.1107/S0021889803000268 27630371 PMC5023043

[B59] Winn M. D. Murshudov G. N. Papiz M. Z. (2003). Macromolecular TLS refinement in REFMAC at moderate resolutions. *Methods Enzymol.* 374 300–321. 10.1016/S0076-6879(03)74014-2 14696379

[B60] Wu H. Min J. Ikeguchi Y. Zeng H. Dong A. Loppnau P. (2007). Structure and mechanism of spermidine synthases. *Biochemistry* 46 8331–8339. 10.1021/bi602498k 17585781

[B61] Zhou X. Chua T. K. Tkaczuk K. L. Bujnicki J. M. Sivaraman J. (2010). The crystal structure of *Escherichia coli* spermidine synthase SpeE reveals a unique substrate-binding pocket. *J. Struct. Biol.* 169 277–285. 10.1016/j.jsb.2009.12.024 20051267

